# Harold Frederic and Christian Science

**Published:** 1898-12

**Authors:** 


**Monthly Review of Medicine and Surgery.**

*EDITORS:*

THOMAS LOTHROP, M. D. - - WM. WARREN POTTER, M. D.

All communications, whether of a literary or business nature, should be addressed to the managing editor: 284 FRANKLIN STREET, BUFFALO, N. Y.

VOL. XXXVIII. DECEMBER, 1898. NO. 5.

### HAROLD FREDERIC AND CHRISTIAN SCIENCE.

THE death of Harold Frederic, the literateur and newspaper correspondent, which occurred in London, October 19, 1898, while under the care of some of the apostles of Christian science, so-called, has occasioned a profound sensation, not only in England, but throughout the United States where the distinguished writer was well known and had many friends. The circumstances attending his sickness and death are briefly as follows: he had been in poor health for some time, but it was not believed that the malady was necessarily a fatal one, though its precise nature does not appear. He was attended by a regular physician for a time, who made a favorable prognosis. Soon, however, he was influenced to dismiss his physician and employ a christian science healer who, it is needless to add, was a woman. She "threw medicine to the dogs," completely changed the whole management of the case, and substituted the empty nothingness of the fake healer for the substantial ministrations of scientific medicine. Soon afterward Mr. Frederic died and the persons responsible for the change and who pretended to minister to him were arrested. A London despatch, dated November 8, 1898, says:

> The coroner's jury which has been investigating the death, on October 19th last, of Harold Frederic, the late correspondent of *The New York Times*, rendered a verdict today of manslaughter against Kate Lyon, a member of the late Mr. Frederic's household, and Mrs. Mills, a Christian scientist.

Mr. Frederic was born at Utica, N. Y., August 19, 1856. He received his early education in the common schools of Utica, and began his lifework as a draughtsman, next became a reporter, then editor of the *Utica Observer*, afterward editor of the *Albany*

*Journal* and went to Europe in 1884 as correspondent of the *New York Times*, in which work he continued until his death. He became noted in the world of fiction as a graphic and forceful writer and published many books and sketches.

Frederic's most important newspaper work was done in connection with the cholera in Southern France and Italy. He told the cause of that disease and described the heroism of the sisters. Subsequently he went to Russia and wrote up the persecution of the Jews, for which he was practically driven out of that country and had never been able to return. He described also the funerals of the Emperor Frederick and of President Carnot.

That such an intelligent and well-informed man should become a believer, follower and victim of such hollow mockery as the misnamed medical fake, known as christian science, is only another illustration of the well-known fact that even great minds have their equally great weaknesses. It is only a step from the sublime to the ridiculous. That such a famous man should influence others to follow his lead is only natural, and but for the fact that the British law courts will step in to interpret the system in this instance and place it before the people at its true value, no doubt many converts to this fetich would follow this remarkable espousal of a medical religion.

The chief interest in this matter now centers around the possible legal outcome at the trial. English law usually is not slow to apprehend or punish criminals. In the United States it seems difficult to obtain convictions in similar cases. The sympathy of a jury and the weakness of the bench often combine to render adequate punishment a rare sequel. In Kentucky the practice of medicine has been well defined to be any ministration that pretends to offer to cure bodily ills for pay. But, too often, either the court itself or some of the jurors fancy they have been benefited by christian science, hence convictions are not easy. Even lawyers of reputation for intelligence on ordinary subjects become believers in this weak combination of medicine and religion, and are willing advocates of the "oppressed" defendant.

We shall watch the progress of the prosecution of these "Sisters" in the Frederic case with much interest, not doubting the ability of the English courts to mete out swift and adequate justice.

---

DR. NICHOLAS SENN, sometime Lieutenant Colonel U. S. Volunteers and Chief of the Operating Staff of the Armies in the field, complained that he did not receive more than two letters in Cuba out of more than 200 written to him, the inference being that the department was at fault.

From a report of the operations of our postal service in and around Santiago, Cuba, that has lately been submitted to First Assistant Postmaster-General Heath by Postal Agent Louis Kempner, in charge at Santiago, we learn some interesting facts bearing on the subject, and showing that the government had done its whole duty in attempting to establish an army mail service.

Mr. Kempner shows that on the 23d of June, before the landing of General's Shafter's invading army had been half accomplished, agents of the post office department had opened a post office in the abandoned headquarters of the Spanish garrison at Baiquiri. The first postal agent assigned to this duty, Mr. Eben Brewer, laid down his life in the service. On the 26th of June the pioneer army mail from Cuba to the United States was put on board the dispatch boat Dolphin. Three days later nearly 300 sacks of mail matter were delivered at Baiquiri, more than half of it addressed to the volunteer regiments in the Fifth Army Corps, viz. : The Rough Riders, the 71st New York and the 2d Massachusetts Volunteer Infantry, then on their way to the fighting line.

Postal Agent Brewer determined that this mail should be delivered at any cost. Finding it impossible to secure army transportation he bought a horse, hired a Cuban with a pony, loaded both the animals with mail sacks and, with the Cuban for a guide, started through the jungle for the front. He succeeded in delivering his mail and then remained four days longer under fire, aiding in carrying the wounded from the field and in attending them in hospitals.

One hundred and twenty more sacks of mail from the United States were delivered at Siboney on July 10th and four clerks arrived to reinforce the slender postal force, which in the meantime had been slightly augmented by details of enlisted men from the Army, and of this force Mr. Kempner took charge. When the yellow fever infected buildings at Siboney were ordered burned by the military authorities, every piece of mail matter was saved, although the postal employees had to submit to the destruction of their own clothing and personal effects. Prompt measures were taken to disinfect the mails and service was resumed after only a brief interruption.

---

Major A. H. Appell, Surgeon U. S. Army, who is well known in Buffalo, where he was stationed for some years, recently made an official report to the Surgeon General of his observations in Cuba.

He used plain words and placed some things in a different light from that in which they had formerly been viewed. Among other things he said :

While the relief associations did excellent work during the latter part of the war, in the emergency of the campaign at Santiago the medical department of the Army was our sole reliance and it required all the training and experience of the regular medical officers, with the Fifth Corps, few enough in number, to meet the emergency.

There was no lack whatever of necessary medical and surgical supplies. But after the Battle of Quasina when we brought the wounded down the hill at Siboney, they were woefully lacking in change of raiment, having landed with but the clothes they had on their backs, which were torn into rags, covered with mud, saturated in many instances with blood from their wounds.

The steamer State of Texas, chartered and loaded with supplies of all kinds by the Red Cross Association, with Miss Clara Barton on board, about this time came to anchor at Siboney. Accompanied by my executive officer, Lieut. D. C. Howard, I called upon Miss Barton, explained to her the situation and asked her whether she could supply these men with clean underwear and pajamas. The president of the National Red Cross Society received me most cordially, showed me the cargo manifest of the State of Texas, wherein there were listed numerous boxes of clothing, but stated that the supplies were not for the soldiers; it was the government's business to look after them, not the business of the Red Cross and that all the supplies in her charge were for the Cuban reconcentrados and all would be held until it was possible to deliver them to these people.

There were at that time a number of surgeons on board the State of Texas and four trained nurses, but although we were working night and day taking care of the sick and wounded, no assistance was given by them until some days afterwards, when our own men were ready to drop from fatigue.

My mission, so far as the Red Cross ship was concerned, was a failure. The net result was a society tract, which Miss Barton kindly presented to me. As I was leaving the ship I was requested to accept a few bottles of malted milk. About a month later I received a communication from the accountant of the association requesting a receipt for the same as a basis upon which to make claim upon the Government for reimbursement.

---

A GOOD place for christian scientists has at last been found. The Rev. Thomas R. Slicer, pastor of All Souls Church, formerly of the Church of Our Father, Buffalo, responded to the toast, "Our Forefathers," at a dinner given by the society of Mayflower Descendants, on Tuesday night, November 22, 1898, at the Waldorf-Astoria, New York, during the course of which he said :

We have in this country several groups of people who need transplanting. Take the islands of the Philippines. To one of these let us send, for instance, the Single Taxers. There they can begin at the bottom and work out their salvation on virgin soil. On another, with a space of water between, we can put Col. Bryan and his friends. We can probably find one of the islands with a silver deposit. There are martyrs of all kinds in America. Send them on a modern Mayflower to the Philippines. It would give them peace and leave us to enjoy life. Social Democrats, Christian scientists, silverites and all—give them an island apiece and let them grow.

---

THE Cleveland *Journal of Medicine* in its issue for October, 1898, publishes an appropriate apology for the part played by some of its citizens in contributing to the death of Dr. George W. Lindheim. We quote the *Journal's* own words as follows :

We of this city should bow our heads in shame over the death in New York, September 16th, of Dr. George W. Lindheim, a Red Cross surgeon. Early in September he passed through Cleveland in charge of a train having on board the sick of the Eighth New York Volunteers. The superintendent of the Huron Street Hospital and several physicians were at the station, saw the sick on the train and said it was a crime not to put them in hospitals here. Dr. Lindheim said he had them all well in hand, and as they had come so far and were so near home it would be best to continue. For this, our local papers and some physicians criticised him most bitterly and unjustly. Not a man under his care died, as the sequel shows, but heroic overwork and worry over the unjust attacks upon him emanating from this city so undermined his health that he became infected with typhoid fever, and died raving against his unjust accusers. It is no exaggeration to say that Dr. Lindheim was murdered by the sensational journalism of the day.

It is now in order for the Buffalo *Express* to don its sack-cloth-and-ashes suit.

---

THERE are those who will not be convinced no matter what the character of the evidence submitted to disprove their convictions, regarding the conduct of the war or the condition of the military camps. For instance, a few weeks ago General Wheeler testified before the investigating commission that the camp at Montauk—Camp Wikoff—never was surpassed in its appointments, military and medical. On the 17th of November, Col. W. H. Forwood, Assistant Surgeon-General reiterated the statement and gave details showing why this was the case. Among other things he said :

As a matter of fact hospitals never were so abundantly supplied. The army rations were supplemented by everything that could be found in the larder of the Waldorf-Astoria, including roast turkey, pheasants, squabs, lambs, sheep, pigs, game of all kinds, pate de foie gras, mineral waters, the finest champagnes and liquors. At one time he had noticed eight barrels of brandy and a large number of cases of wine. There was so much apollinaris water that it was in the way and when a nurse came for a bottle they would offer her a case.

He said the newspaper men at the camp were gentlemen, but they appeared to be under orders to criticise the camp. He knew of two instances in which the reporters had received orders to "roast everything." He had told them not to spare where they found criticism justified.

In conclusion Dr. Forwood said that many patients, who had been removed by their friends, had died and that in many instances their deaths were due to the kindness of those who had taken them in charge.

Even after such undisputed evidence the newspapers and the people in considerable numbers are still growling.

---

THE Medical Society of the County of Kings laid the corner stone of its new building, now in process of erection, on Grant square, Brooklyn, November 10, 1898. The following order of exercises was observed: Opening of the meeting, Frank E. West, A. M., M. D., chairman of the building committee; invocation, Richard S. Storrs, D. D., LL. D., president of the Long Island Historical Society; The Society's work, George MacNaughton, M. D.; Laying of the corner-stone, Joseph H. Hunt, M. D., president of the society; Address, Seth Low, LL. D., president of Columbia College; Benediction, Rev. Father Malone, S. T. D., regent of the University of the State of New York. The society is to be congratulated upon its progress.

---

THE *Richmond Journal of Practice* in its issue for November, 1898, asks the question: shall it be "surgical interference" or "surgical intervention?" and then proceeds to answer it very appropriately in the following manner:

We have never understood why authorities in surgery use the word "interference" when speaking of operative or surgical treatment. When a surgeon performs an operation for the correction of a deformity, the mitigation of pain or the saving of life, does he mean to say that he interferes? If it be interference, then he is culpable; but certainly no operator will plead guilty to the charge of doing meddlesome surgery, and the inevitable conclusion is that the term "surgical interference" is a misnomer. Whenever we read it in text-books or in current literature, we feel like substituting the word *intervention* for "interference," using the word intervention in the sense of interposition, or better still, mediation—a coming between for a friendly purpose. The word interference suggests the idea of collision, clashing, opposition, officiousness, intermeddling, etc.

According to Webster: "A man may often *interpose* with propriety in the concern of others; he can never *intermeddle* without being impertinent or officious; nor can he *interfere* without being liable to the same charge, unless he has rights which are interfered with."

Let us see what Trench has to say. We quote: "In our practical use, *interference* is something offensive. It is the pushing in of himself between two parties on the part of a third who was not asked, and is not thanked for his pains, and who, as the feeling of the word implies, has no business there; while interposition is employed to express the friendly, peacemaking mediation of one whom the act well became, and who, even if he was not specially invited thereunto, is still thanked for what he has done."

A few days ago we suggested this improved phraseology to two of our surgical friends, both of whom are teachers of surgery and liberal contributors to surgical literature. They agreed with us that the point was well taken, and announced it as their intention to adopt the suggestion. Speaking for ourselves, this journal will hereafter use the term surgical *intervention* instead of surgical interference, and we shall hope to see its general adoption by surgical writers.

---

A MEMORIAL to Dr. John Blair Gibbs, in the form of a tablet, was unveiled at Rutgers College, New Brunswick, N. J., November 10, 1898. Dr. Gibbs, who was serving with the Marine Corps at Guantanimo, it will be remembered was the first medical officer killed in action during the war with Spain. The tablet was presented by Professor Robert W. Prentice, of Rutgers, a classmate of Dr. Gibbs, on behalf of the class of '78, which erected the tablet, and the principal address was most appropriately made by Surgeon General William Van Reypen, United States Navy.

The death of Dr. Gibbs has been further commemorated by naming the hospital at Camp Hamilton, Lexington, Ky., the John Blair Gibbs Hospital. The trustees of Mount Lebanon Hospital at New York have also erected a tablet to his memory.

---

THE New York Chamber of Commerce most appropriately has decided to create a fund of $100,000 as a memorial to Colonel George E. Waring, Jr. The income of this fund will go to Mrs. Waring, and at her death a permanent memorial, perhaps a hospital, will be established in testimony of Col. Waring's sterling worth and integrity as a citizen, and for the conspicuous services he rendered the country and especially to the City of New York.

---

THE first honest newspaper correspondent to appear before the War Investigation Commission seems to be Mr. James J. F. Archibald, a San Francisco newspaper man, who was with the First Regular

Infantry throughout the war and who testified, November 23d, as to conditions in Cuba. He said he had volunteered to appear before the commission to correct some published statements regarding hardships resulting from lack of food. He had much experience campaigning with the regular troops in the west and they had always had a harder time out there than they did before Santiago. The only reason the Volunteers suffered was because of their inexperience. They did not know how to get out and hustle for rations.

All honor to Mr. Archibald. Now let some of the newspapers so anxious to publish all that bears on the other side give editorial prominence to this paragraph.

